# Supplementary material for: An ingestible light source for deep photoacoustic imaging
Source: Photoacoustics. 2026 Jun 2;50:100844. doi: 10.1016/j.pacs.2026.100844 (PMC13264102; doi:10.1016/j.pacs.2026.100844)
Supplement: Supplementary file 1 — Supplementary material [file mmc1.docx]

Supplementary Information for:

An Ingestible Light Source for Deep Photoacoustic Imaging

David C. Garrett and Lihong V. Wang

*Caltech Optical Imaging Laboratory, Andrew and Peggy Cherng Department of Medical Engineering, Department of Electrical Engineering, California Institute of Technology, Pasadena, CA 91125, USA*

* Corresponding author: lvw@caltech.edu

**Sensitivity analysis**

The optical energy of $\sim130 \mu J$ used here is far lower than that in typical PAT systems, but we illuminate a narrower region near the laser. Here, we theoretically estimate the SNR of this system. While the fluence in this system depends on the target distance from the laser surface, we approximate it here as $F\sim130 \mu J/cm^{2}$. For our pulse width of $\Delta t=0.6 \mu s$, photoacoustic stress confinement is valid for imaging absorbers with characteristic size $d>\Delta tc_{s}\sim0.9 \mathrm{mm}$, where $c_{s}\sim1540 m/s$ is the speed of sound in tissue. The resulting initial photoacoustic pressure is $p_{0}=\Gamma\mu_{a}F$ [1]. For tissue, we consider $\mu_{a}\sim0.1 \mathrm{cm}^{-1}$, and for the wire target we approximate $\mu_{a}\sim2.0 \mathrm{cm}^{-1}$. The Grüneisen coefficient $\Gamma$ is $\sim0.2$ (dimensionless). This results in an initial pressure $p_{0,\mathrm{tissue}}\sim2.6 \mathrm{Pa}$ and $p_{0,\mathrm{wire}}\sim52 \mathrm{Pa}$.

We then compare this pressure with the noise-equivalent pressure (NEP) of our system [2]. We consider an NEP spectral density of 0.5 $\mathrm{mPa}\mathrm{Hz}^{-1/2}$ of each acoustic receiver element [3], corresponding to $\mathrm{NEP}_{0}$ as 0.5 Pa over a 1 MHz bandwidth. The NEP of a single recorded receiver channel $\mathrm{NE}P_{\mathrm{sig}}$ can be estimated by averaging over the number of shots $N_{\mathrm{avg}}$ and by scaling the NEP from the receiver surface to the target location $r$. In our human-scale system, $r\sim30 \mathrm{cm}$. We consider the acoustic wavelength at 1 MHz, and our experiments used $N_{\mathrm{avg}}=2000$.

|  | $\mathrm{NEP}_{\mathrm{sig}}=\mathrm{NEP}_{0}\frac{r/\lambda}{\sqrt{N_{\mathrm{avg}}}}\sim2.2 \mathrm{Pa}$ | (1) |
| --- | --- | --- |

This would result in a signal-to-noise ratio (SNR) of $p_{0,\mathrm{wire}}/\mathrm{NEP}_{\mathrm{sig}}\sim24$. In the recorded signals shown in Supplementary Figure 1, we observe an SNR of approximately 27. Note that these signals were notch filtered at 500 kHz to remove residual signals from acoustic power transfer, but some ringing remains.

The resulting NEP of the entire imaging system can be estimated [3] by also averaging over the receiver element count $N_{\mathrm{ele}}=512$. The resulting imaging $\mathrm{NEP}_{\mathrm{img}}$ is estimated as

|  | $\mathrm{NEP}_{\mathrm{img}}=\mathrm{NEP}_{0}\frac{r/\lambda}{\sqrt{N_{\mathrm{avg}}}\sqrt{N_{\mathrm{ele}}}}\sim0.1 \mathrm{Pa}$ | (2) |
| --- | --- | --- |

In realistic tissues with $\mu_{a}\sim0.1 \mathrm{cm}^{-1}$, we expect the resulting image SNR to be approximately:

|  | $\mathrm{SNR}_{\mathrm{img}}=\frac{p_{0,\mathrm{tissue}}}{\mathrm{NEP}_{\mathrm{img}}}\sim26$ | (3) |
| --- | --- | --- |

**Supplementary Figure 1. Example recorded signals using WPAT excitation**. **a**, Recorded sinogram across all 512 channels after notch filtering the 500 kHz acoustic power transfer signal. **b**, Example signal on channel 310.

We calculated the image SNR using a single device position without Gaussian windowing to preserve the noise in the background. The SNR was found as ~17.

The frequency spectrum of an example recorded signal is shown in Supplementary Figure 2 before and after filtering. Note that the dominant photoacoustic response lies above the notch filter frequency of 500 kHz. We also apply a bandpass filter between 0.3 – 2.0 MHz.

**Supplementary Figure 2. Frequency spectrum of recorded signals before and after filtering.**

**Image generation**

We raster-scanned the WPAT device to generate a cm-scale image of a wire target. For each position, we reconstruct the photoacoustic image. We apply a Gaussian window with $\sigma=6 \mathrm{mm}$ centered at the device position to isolate the illuminated imaging region while minimizing the inclusion of image noise. An example window and image for a single position is shown in Supplementary Figure 3. The final image is the sum of the images from all 50 positions.

**Supplementary Figure 3. Example image from a single device location during raster-scanning**. **a**, Gaussian window used to isolate the illuminated region. **b**, Photoacoustic image after applying the Gaussian window. The final image is generated by summing the windowed images from all device positions.

The expected resolution is shown as ~0.9 mm in Supplementary Figure 4, where the phantom’s wires are < 0.5 mm in thickness.

**Supplementary Figure 4. Resolution assessment for the WPAT system. a,** Phantom image synthesized from all device positions. **b,** Extracted image profile along the cyan line in **a**. The dashed line shows the real signal, and the blue line shows the envelope. The full width at half-maximum (FWHM) is found as ~0.9 mm.

**Acoustic power transfer beam profiles**

We use a flat 1.5-inch diameter transmitter to power the WPAT device. This geometry corresponds to a Rayleigh length of ~12 cm. Compared with focused transmitters, flat ones provide a more uniform focal region with reduced positioning precision requirements. The simulated beam profile of this transducer is shown in Supplementary Figure 5.

Supplementary Figure 5d also shows the simulated on-axis normalized power transfer between the transmitting element and the 6 mm diameter receiver. Note that 80% of peak power transfer is maintained over a ~8 cm axial distance, which relaxes positioning accuracy requirements in our experiments.

**Supplementary Figure 5. Simulated acoustic beam profile for acoustic power transfer**. **a**, Simulated beam profile for the transmitter. **b**, Transverse profile of the normalized pressure at the Rayleigh length of 12 cm. The red-dashed lines indicate the diameter of the receiving transducer. **c**, Simulated on-axis normalized pressure. **d**, Simulated on-axis power transferred to a receiving 6 mm diameter element.

We also model the effect of receiver tilt angle on received electrical power in Supplementary Figure 6. As shown, modest tilt angles sharply reduce received power, consistent with prior work [4]. This sensitivity to angular misalignment poses a practical challenge in the GI tract, where the capsule orientation shifts continuously during transit. Future designs could address this through omnidirectional receiver geometries, such as conformal transducers wrapped around the capsule housing, or through an array-based transmitter capable of dynamic beam steering to maintain optimal acoustic coupling regardless of capsule orientation.

**Supplementary Figure 6. Simulated received power with varying receiver tilt angle.**

We show the rectified DC voltage from acoustic power transfer to the capacitor bank in Supplementary Figure 7a. Laser firing causes a sharp drop in voltage from ~15 V to 9 V, corresponding to an energy of $E_{LD}=\frac{1}{2}C\left( V_{i}^{2}-V_{f}^{2} \right)\sim324 \mu J$. Note that this example is shown for 100 Hz laser repetition rate. We show a magnified view of the bus voltage during laser firing in Supplementary Figure 7b, where there is a slight (~0.5 V_pp_) ringing at ~2 MHz owing to the residual inductance between the capacitor bank and laser diode.

**Supplementary Figure 7. Example rectified voltage from the acoustic receiver to the capacitor bank used to energize the pulsed laser diode array**. **a,** Acoustic charging of the bus voltage (V_bus_). The laser is fired here every 10 ms. **b,** Magnified view of the bus voltage waveform during laser firing.

**Thermal safety analysis**

The total thermal power dissipated within the sealed capsule was estimated analytically from the device power budget. The acoustic source delivers ~200 mW incident on the capsule, of which ~16 mW is converted to electrical power used by the laser diode and ~5 mW is converted to optical power. Of the incident acoustic power on the capsule, some of that power is reflected back into the acoustic medium. To provide a conservative thermal estimation here, we assume the entire 200 mW incident acoustic energy is resistively dissipated as heat in the device.

To estimate the resulting surface temperature rise, we model the capsule as a finite cylinder (26 × 11 mm, surface area A ~ 9.5 cm²) dissipating 200 mW uniformly into the surrounding medium. Using a conservative convective heat transfer coefficient for the GI lumen of h = 200 W/m²·K (stagnant fluid, no perfusion), the steady-state surface temperature rise is:

$$\Delta T=\frac{Q}{h\times A}=\frac{0.2 [W]}{200 \times9.5}\approx0.11 ^{\circ}C$$

This is well within the ≤ 2 °C safety limit specified for ingestible/implantable devices (ISO 14708-1). The estimate is conservative: h = 200 W/m²·K assumes a stagnant medium, whereas GI peristalsis and fluid movement will increase convective cooling. Furthermore, the pulsed operating mode means instantaneous heat generation is lower than the steady-state assumption used here.

**Device construction**

Steps of the device construction are shown in Supplementary Figure 8. The front surface of the 3D printed capsule is first coated with PDMS. The wired device, including the receiving transducer, matching network, and the three PCB modules, are then positioned into the capsule. The two arms of the antenna are connected to one of the PCBs. After insertion into the device, the rear face of the receiving transducer is also coated in a thin layer of PDMS.

**Supplementary Figure 8. Device construction**. **a**, Wired device before inserting into the 3D-printed enclosure. **b**, Front face of the device with the laser diode array. **c**, Rear face of the device with the receiving transducer. Both faces are coated with PDMS.

**References**

[1] L. V. Wang and H. Wu, *Biomedical optics: principles and imaging*. John Wiley & Sons, 2012.

[2] A. M. Winkler, K. Maslov, and L. V. Wang, “Noise-equivalent sensitivity of photoacoustics,” *J. Biomed. Opt.*, vol. 18, no. 9, p. 097003, Sep. 2013, doi: 10.1117/1.JBO.18.9.097003.

[3] D. C. Garrett and L. V. Wang, “Acoustic sensing with light,” *Nat. Photonics*, vol. 15, no. 5, pp. 324–326, May 2021, doi: 10.1038/s41566-021-00804-z.

[4] H. Basaeri, Y. Yu, D. Young, and S. Roundy, “Acoustic power transfer for biomedical implants using piezoelectric receivers: effects of misalignment and misorientation,” *J. Micromechanics Microengineering*, vol. 29, no. 8, p. 084004, Jun. 2019, doi: 10.1088/1361-6439/ab257f.
